# Localization and Tissue Tropism of Ostreid Herpesvirus 1 in Blood Clam *Anadara broughtonii*

**DOI:** 10.3390/biology13090720

**Published:** 2024-09-13

**Authors:** Ya-Nan Li, Xiang Zhang, Bo-Wen Huang, Lu-Sheng Xin, Chong-Ming Wang, Chang-Ming Bai

**Affiliations:** 1 College of Ocean and Biology Engineering, Yancheng Teachers University, Yancheng 224007, China; liyn@yctu.edu.cn; 2 Key Laboratory of Maricultural Organism Disease Control, Ministry of Agriculture, Qingdao Key Laboratory of Mariculture Epidemiology and Biosecurity, Yellow Sea Fisheries Research Institute, Chinese Academy of Fishery Sciences, Qingdao 266071, China; zhxiang1997@126.com (X.Z.); huangbw@ysfri.ac.cn (B.-W.H.); xinls@ysfri.ac.cn (L.-S.X.); wangcm@ysfri.ac.cn (C.-M.W.); 3 Laboratory for Marine Fisheries Science and Food Production Processes, Qingdao National Laboratory for Marine Science and Technology, Qingdao 266237, China

**Keywords:** OsHV-1, *Anadara broughtonii*, in situ hybridization, tissue tropism, hemocytes

## Abstract

**Simple Summary:**

OsHV-1 is one of two herpesviruses known to infect invertebrates; this pathogen has emerged as the primary etiology responsible for mass mortalities in the species. The pathological characteristics, tissue and cellular tropisms of OsHV-1 in *Anadara broughtonii* (*A. broughtonii*) remain unknown. The objective of this study was to characterize the pathological changes and tissue tropism during the development of an OsHV-1 infection in *A. broughtonii*. The results demonstrated that hemocytes and fibroblastic-like cells were the primary cellular targets of OsHV-1. Additionally, lesions, infiltrated hemocytes, and co-localized ISH signals were identified in the muscular tissues of the foot and adductor muscle. These findings contribute to the understanding of OsHV-1 pathogenesis in Arcidae mollusks.

**Abstract:**

OsHV-1 caused detrimental infections in a variety of bivalve species of major importance to aquaculture worldwide. Since 2012, there has been a notable increase in the frequency of mass mortality events of the blood clam associated with OsHV-1 infection. The pathological characteristics, tissue and cellular tropisms of OsHV-1 in *A. broughtonii* remain unknown. In this study, we sought to investigate the distribution of OsHV-1 in five different organs (mantle, hepatopancreas, gill, foot, and adductor muscle) of *A. broughtonii* by quantitative PCR, histopathology and in situ hybridization (ISH), to obtain insight into the progression of the viral infection. Our results indicated a continuous increase in viral loads with the progression of OsHV-1 infection, reaching a peak at 48 h or 72 h post-infection according to different tissues. Tissue damage and necrosis, as well as colocalized OsHV-1 ISH signals, were observed primarily in the connective tissues of various organs and gills. Additionally, minor tissue damage accompanied by relatively weak ISH signals was detected in the foot and adductor muscle, which were filled with muscle tissue. The predominant cell types labeled by ISH signals were infiltrated hemocytes, fibroblastic-like cells, and flat cells in the gill filaments. These results collectively illustrated the progressive alterations in pathological confusion and OsHV-1 distribution in *A. broughtonii*, which represent most of the possible responses of cells and tissues to the virus.

## 1. Introduction

The initial case of a herpes-like virus infection in mollusks was documented in *Crassostrea virginica* in the USA in 1972, representing the inaugural herpesvirus infection in invertebrate animals [1]. Following such a discovery, other potential herpesvirus infections were reported in *Ostrea edulis* and *Mercenaria mercenaria* in the UK [2]. These cases were confined to local populations, and received little attention from the aquaculture sector. However, mass mortalities of hatchery-reared larval *Crassostrea gigas* associated with herpesvirus infection were reported in France and New Zealand in the summer of 1991 [3,4]. The genomic sequence and capsid structure of the virions purified from infected *C. gigas* larvae were subsequently resolved [5]. These results collectively led to the formal taxonomy of Ostreid herpesvirus 1 (OsHV-1) infecting *C. gigas*, and established a new family (Malacoherpesviridae) under the umbrella of the order Herpesvirales to incorporate invertebrate herpesviruses [6]. While the homology between OsHV-1 and the mollusk herpesviruses discovered in the 1970s cannot be currently confirmed, since 1991, mass mortality events associated with herpesvirus-like particles have been observed in a variety of bivalve species from a dozen of countries and regions worldwide [7]. In addition to *C. gigas*, OsHV-1 infections have been associated with mortalities of other bivalves, including oysters, scallops, and clams [8]. More recently, OsHV-1 infection was also detected in a crustacean (*Carcinus maenas*) and cephalopod (*Octopus vulgaris*) by polymerase chain reaction (PCR) and ISH [9,10]. 

The production of mollusks in aquaculture has increased exponentially in recent decades, with China accounting for most of this growth [11]. In 2021, global marine mollusk aquaculture production constituted 31.0% of the total global marine aquaculture production by weight [12]. In China, the ratio was 69.0% [13]. The blood clam, *Anadara broughtonii*, previously known as *Scapharca broughtonii*, is member of the phylum Mollusca, class Bivalvia, order Arcida, and family Arcidae [14,15]. *A. broughtonii* is distributed along the Pacific Northwest coastline covering China, Japan, Korea, and the Far Eastern part of Russia. *A. broughtonii* burrows shallowly in sandy mud or muddy bottoms at 5–50 m depths [16]. The name “bloody clam” originated from the red color of their visceral mass, which is due to the presence of hemoglobin in both tissues and hemolymph [14]. Adult blood clams can reach a shell length of 100 mm, which are characterized by thick and harder calcareous shells, covered by a hairy brown periostracum [16]. This species is harvested as a source of sashimi, which has resulted in intensive fishing and a significant decline of the wild resources, particularly after the early 1990s [17,18]. Many efforts have been made to recover the wild population stocks of *A broughtonii* in China, Japan, and Korea [19,20]. In China, a large amount of *A. broughtonii* seeds were produced in hatchery and cultured in Bohai and the Yellow Seas of China [17]. Such aquaculture and stock enrichment practices have revealed the susceptibility of *A. broughtonii* to many pathogenic bacteria and viruses, including a variant of OsHV-1 [20,21,22]. The first documented instance of mass mortalities of *A. broughtonii* associated with OsHV-1 infection was observed in hatchery-reared broodstocks in Northern China in 2012 [22,23]. The complete genome sequence of the OsHV-1 variant infecting *A. broughtonii* has been determined [24,25]. Additionally, the transcriptional changes and potential immune responses of *A. broughtonii* against OsHV-1 during the progression of OsHV-1 infection have been investigated [26]. Moreover, temperature and elemental iron have been identified as crucial external and internal factors in the pathogenicity of OsHV-1, respectively [27,28]. Nevertheless, the morphological features and their association with the viral infection, which could provide insight into the understanding the underlying mechanisms of disease, have not yet been subject to rigorous study.

The primary objective of the present study was to characterize the histological features at different time points during an experimental infection of OsHV-1 in *A. broughtonii*. Secondly, the aim was to determine the tissue and cellular tropisms of OsHV-1 at different infection stages. The results may provide insights into the entry and distribution routes of OsHV-1 in *A. broughtonii*, as well as potential cell and tissue responses to the viral infection.

## 2. Materials and Methods

### 2.1. Blood Clams and Acclimation

The blood clams (*Anadara broughtonii*) were obtained from Haichang Aquatic Food Co., Ltd., Qingdao, China. The clams exhibited no clinical signs of illness, with an average weight of 93.22 g, a shell length of 64.5 mm, and a shell height of 51.2 mm. The blood clams were cultivated in eight 60 L aerated tanks, with about 25 clams per tank. The blood clams were initially cultured with sand-filtered seawater at a temperature of 18.0–20.0 °C for a period of two weeks, with the sand-filtered seawater being replaced daily. In the laboratory setting, the blood clams were fed with seaweed (*Laminaria japonica*). Subsequently, 18 individuals were randomly selected for quantitative polymerase chain reaction (qPCR) analysis, which yielded negative results for OsHV-1. The relevant experiments were approved by the local animal care and use committee and conducted in accordance with local and central government regulations. 

### 2.2. Virus Preparation 

The OsHV-1 virus suspension was prepared from the OsHV-1 infected blood clams (stored at −80 °C) collected from Jimo in September 2017. The protocol for virus suspension was employed in accordance with the previously described tissue homogenates method [23]. In brief, the mantle tissues from blood clams with OsHV-1 infection were dissected and homogenized. After a short centrifugation, the supernatant of the mantle homogenate underwent a series of filtration processes employing syringe filters with pore sizes of 5 µm, 2 µm, 0.45 µm, and 0.22 µm, in a succession steps. To serve as a control, mantle tissues from healthy blood clams were used to make negative tissue homogenates in parallel with the samples from infective calms. The filtered mantle homogenate was temporarily stored in an ice bath until required for use. A 200 μL aliquot of the tissue homogenates was utilized for DNA extraction and OsHV-1 DNA quantification. 

### 2.3. Experimental Infection and Sample Collection

#### 2.3.1. Experimental Design

One hundred and eighty (180) blood clams were randomly divided into an infected group (120 animals) and a control group (60 animals). For the infected group, the 120 clams were allocated to six tanks (each containing approximately 50 L of sea water and 20 animals); three tanks were assigned for sampling at different time point, and the other three were employed for mortality surveillance. For the negative control group, the 60 clams were separated into three tanks (each containing approximately 50 L of seawater and 20 animals) for monitoring mortality.

#### 2.3.2. Virus Inoculation and Sample Collection

For the challenged group, 200 μL tissue homogenates (~5 × 10^4^ copies of viral DNA/μL) were injected into the foot of each animal. Meanwhile, each animal in the control group received an injection with an equal volume of the negative tissue homogenate. At the end of the experimental infection, all clams were sacrificed for qPCR analysis. Additionally, six clams from the negative control tank were utilized for histological and ISH examination. During the infection process, one clam was sampled from each of the three challenged tanks for sampling at each time point, namely 0, 6, 12, 24, 48, and 72 (hpi). Cross-sections of clams, including five organs (mantle, gill, hepatopancreas, adductor muscle, and foot), were sampled from each individual. One section was fixed in Davidson’s alcohol-formalin-acetic acid fixative for 24–36 h, after which it was transferred to 70% ethanol in anticipation of a histological and ISH examination. The remaining sections were promptly stored at a temperature of −40 °C for subsequent DNA extraction and qPCR assay. 

### 2.4. DNA Extraction and qPCR Analysis 

The detection and quantification of OsHV-1 DNA were performed by qPCR according to the methods described in a previous report [29]. Total DNA was extracted from five tissue samples of each collected clam as previously described, using the TIANamp™ Marine Animal DNA Kit (TIANGEN, Beijing, China) in accordance with the instructions provided. The concentration and quality of extracted total DNA were determined using a NanoDrop 2000 spectrophotometer (Thermo Scientific^®^, Waltham, MA, USA). qPCR was performed on a Bio-Rad CFX Connect RealTime system (Bio-Rad Laboratories, Hercules, CA, USA). Amplification was conducted in a 25 μL reaction system containing 2 μL DNA, 1 μL of each primer (BF: 5′-GTCGCATCTTTGGATTTAACAA-3′ and B4: 5′-ACTGGGATCCGACTGACAAC-3′). The reaction mixture consisted of 12.5 μL 2 × FastStart Essential DNA Probes Master, 0.5 μL TaqMan^®^ probes (5′-FAM-TGCCCCTGTCATCTTGAGGTATAGACAATC-BHQ-3′) and 8 μL ddH_2_O. The reaction mixture was incubated at 95 °C for 10 min, after which 40 cycles of 10 s at 95 °C and 30 s at 60 °C were performed. The data were expressed as the number of OsHV-1 copies per nanogram of total tissue DNA for the three replicates.

### 2.5. Histology and In Situ Hybridization

The histological sections were made according to standard protocols, including dehydration in ethanol series, clearing, embedding in paraffin and cutting into 3–5 µm thick sections using a rotary microtome. The tissue sections were subjected to staining with hematoxylin and eosin (for histopathological analysis) or treatment with the C2/C6 probe (for ISH). The C2/C6 DNA probe was prepared by incorporating digoxigenin-11-dUTP into PCR products as previously reported [30]. The ISH procedure was a modification of previously published protocols [31,32], and fully described in Bai et al. (2020) [33]. 

The non-specific binding of the antibody was assessed by performing a hybridization of the positive section using hybridization buffer without a DIG-labeled probe. To assess the potential for non-specific staining due to the presence of endogenous alkaline phosphatase, the anti-DIG alkaline phosphatase conjugate was omitted during the testing of the positive sections. Sections were considered positive for OsHV-1 DNA if typical blue/black precipitates were visible within the cells and all controls yielded the expected results.

## 3. Results

### 3.1. Clinical Signs and Mortality 

**Figure 1:**
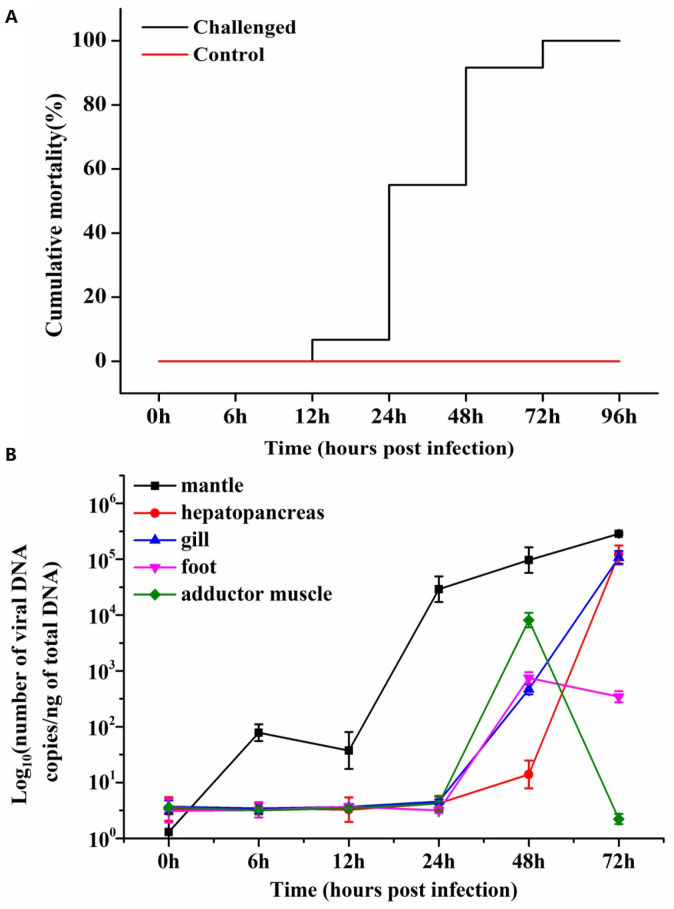
(**A**) The cumulative mortality of *Anadara broughtonii* during experimental infection. The experimental group was injected with OsHV-1 (200 μL of ~5 × 10^4^ copies viral DNA/μL) into the foot of each clam, and the control group with an equal volume of the negative tissue homogenate. (**B**) OsHV-1 viral DNA detection curves by qPCR in different tissues of the blood clam during the infection period.

The survival of blood clams was monitored on a daily basis, and the resulting survival curves were plotted (Figure 1A). The most striking anomaly observed in the experimental group was the turbid and frothy water, which manifested approximately 24 hpi. The principal indication of moribund individuals was the loss of the shell’s capacity to close and the pale visceral mass. The gill filaments exhibited redness and swelling due to the repletion of the gill hemolymph vessels with hemocytes containing hemoglobin. Acute mortality was observed in blood clams at 48 hpi. The cumulative mortality of blood clams was 0% at 6 hpi and 12 hpi, 5% at 24 hpi, 55% at 48 hpi, 95% at 72 hpi and 100% at 96 hpi (Figure 1A). The control group demonstrated no mortality during the viral infection period, and the seawater remained relatively clear.

### 3.2. Viral DNA Quantification by qPCR

The presence of OsHV-1 DNA was first identified in the mantle at 6 hpi. In the foot and adductor muscle tissues, the viral DNA loads peaked at 48 hpi, with amounts of 2.87 × 10^2^ and 3.91 × 10^3^, respectively. However, a subsequent decline was noted at 72 hpi. In the mantle, gill, and hepatopancreas, the viral DNA loads demonstrated a gradual increase throughout the course of infection until 72 hpi (Figure 1B). The viral levels were found to range from 1.13 to 5.45 × 10^5^ DNA copies per ng of mantle DNA; 5.26 to 5.08 × 10⁵ DNA copies per ng of hepatopancreas DNA; 5.70 to 5.03 × 10^5^ DNA copies per ng of gill DNA; 4.94 to 2.87 × 10^2^ DNA copies per ng of foot DNA; and 5.54 to 3.91 × 10^3^ DNA copies per ng of adductor muscle DNA (Table 1). No evidence of OsHV-1 DNA was found in clams from the control group at the end of the experimental infection.

### 3.3. Histopathology and In Situ Hybridization

**Figure 2:**
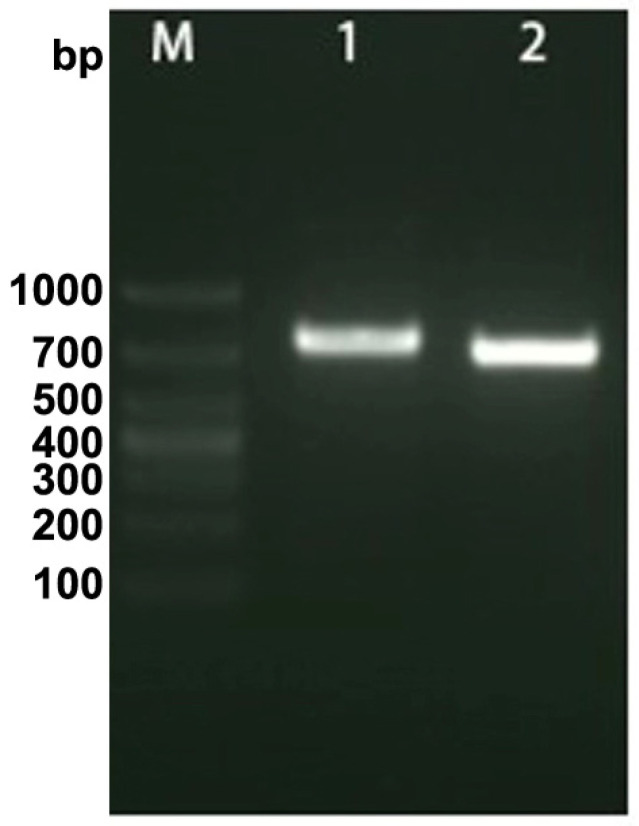
The labeling of OsHV-1 DNA probe with digoxigenin (DIG) -11-dUTP. “M” indicated the DNA markers, “1” and “2” indicated the bands of the DIG-labeled PCR products and the normal PCR, respectively.

The results of agarose gel electrophoresis demonstrated the successful amplification of a fragment of OsHV-1 DNA, as evidenced by the presence of a distinct band with an approximate size of 700 base pairs (bp). Furthermore, the incorporation of DIG was confirmed, resulting in an increase in the molecular mass of the amplified nucleic acid (Figure 2). The original file of Figure 2 was provided in the Supplementary File.

Histological lesions and OsHV-1 DNA signals were not identified in tissue sections of any organs collected during the initial stage of the viral infection. At 48 hpi and 72 hpi, gross lesions and positive signal with the OsHV-1 DNA probe were observed in the hepatopancreas, gill, mantle, foot, and adductor muscle in all cases. No histopathological changes or positive signal were observed in the five tissues of the control group.

**Figure 3:**
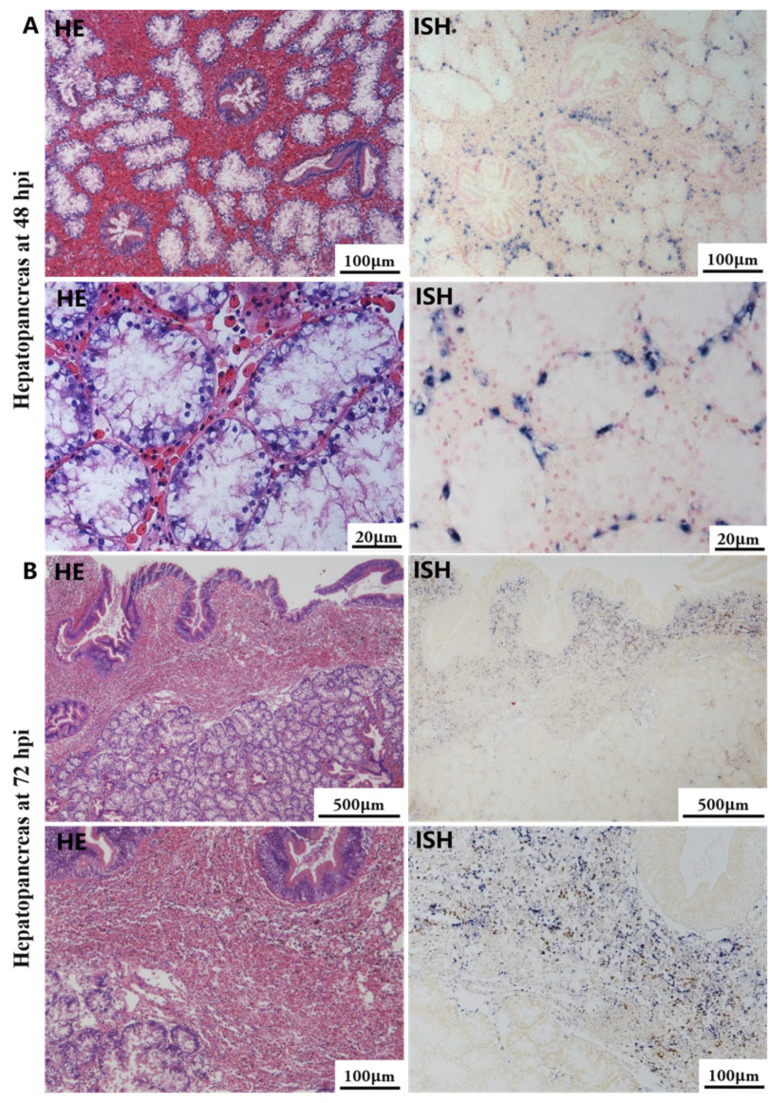
Histopathology and in situ hybridization investigation of OsHV-1 infected *A. broughtonii* hepatopancreas. Positive results are indicated by black/blue precipitates. (**A**) Pathological changes (left) and positive cells (right) in the hepatopancreas at 48 hpi. (**B**) Pathological changes (left) and positive cells (right) in the hepatopancreas at 72 hpi. Scale bar = 500 μm, 100 μm and 20 μm, respectively.

**Figure 4:**
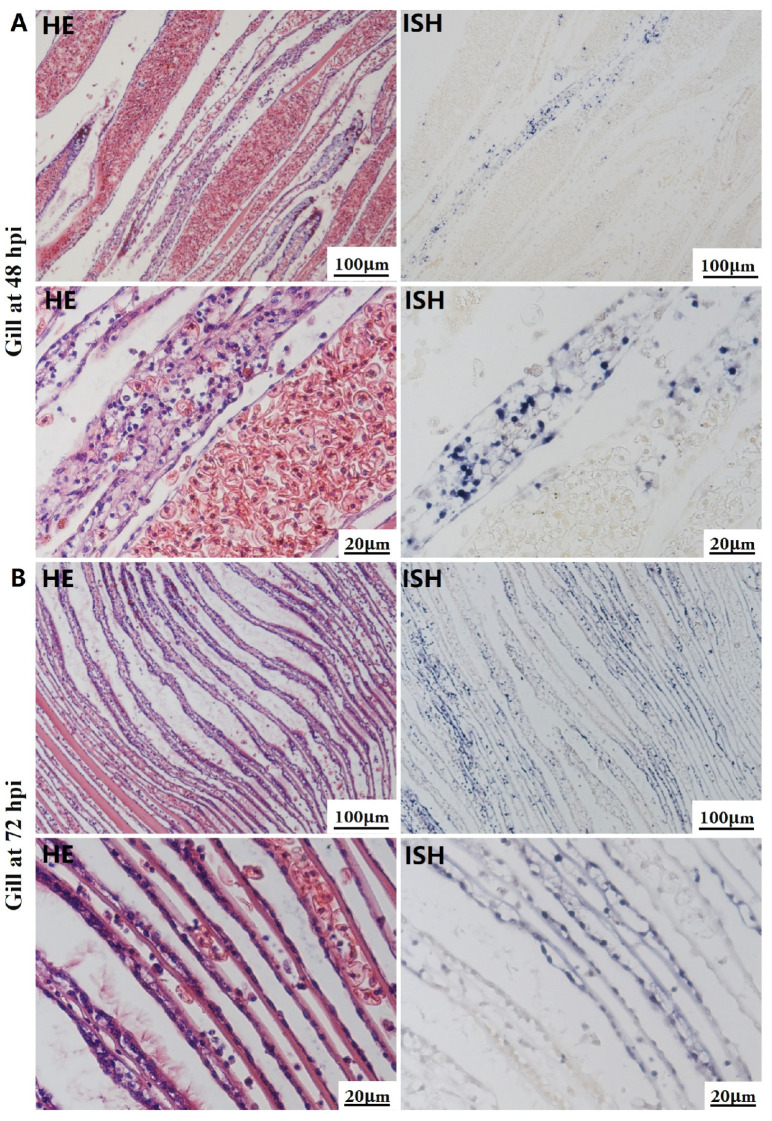
Histopathology and in situ hybridization investigation of OsHV-1 infected *A. broughtonii* gills. Positive results are indicated by black/blue precipitates. (**A**) Pathological changes (left) and positive cells (right) in the gill at 48 h post-injection (hpi). (**B**) Pathological changes (left) and positive cells (right) in the gill at 72 hpi. Scale bar = 100 μm and 20 μm, respectively.

**Figure 5:**
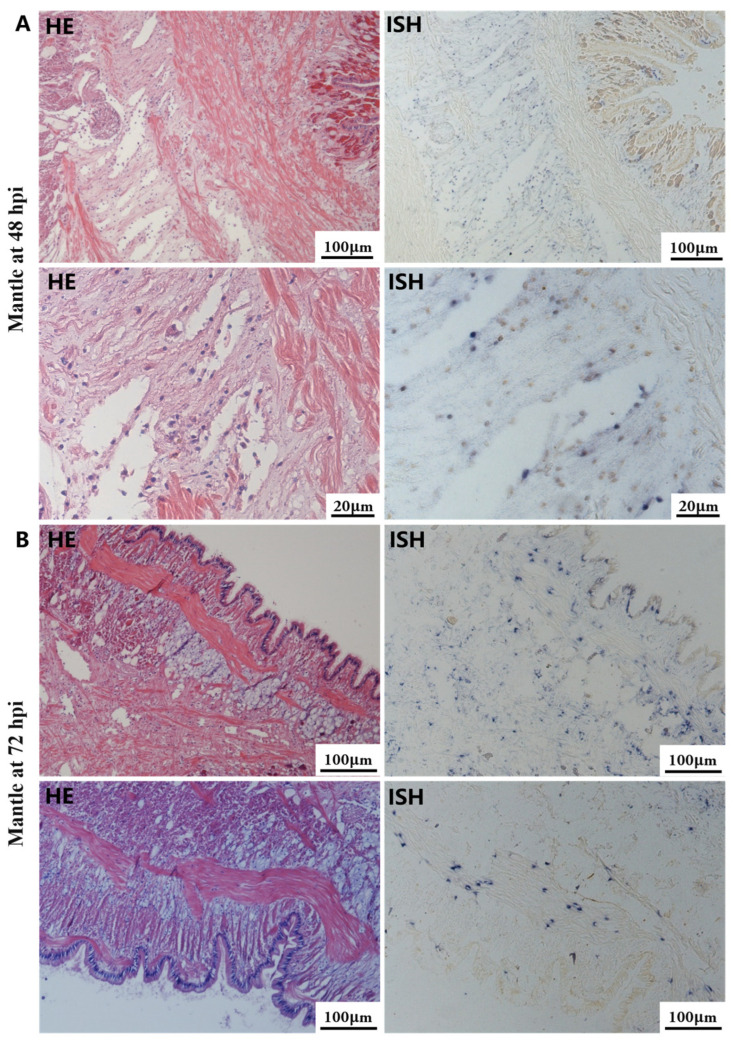
Histopathology and in situ hybridization investigation of OsHV-1 infected *A. broughtonii* mantle. Positive results are indicated by black/blue precipitates. (**A**) Pathological changes (left) and positive cells (right) in the mantle at 48 hpi. (**B**) Pathological changes (left) and positive cells (right) in the mantle at 72 hpi. Scale bar = 100 μm and 20 μm, respectively.

**Figure 6:**
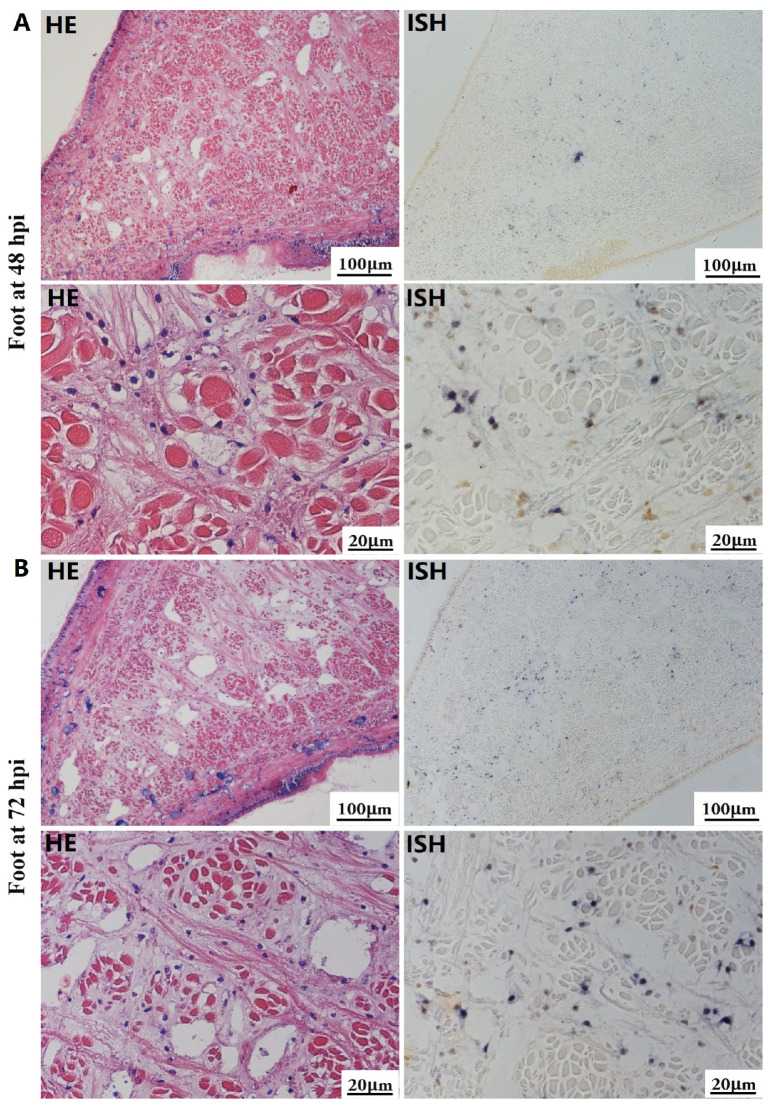
Histopathology and in situ hybridization investigation of OsHV-1 infected *A. broughtonii* foot. Positive results are indicated by black/blue precipitates. (**A**) Pathological changes (left) and positive cells (right) in the foot at 48 hpi. (**B**) Pathological changes (left) and positive cells (right) in the foot at 72 hpi. Scale bar = 100 μm and 20 μm, respectively.

**Figure 7:**
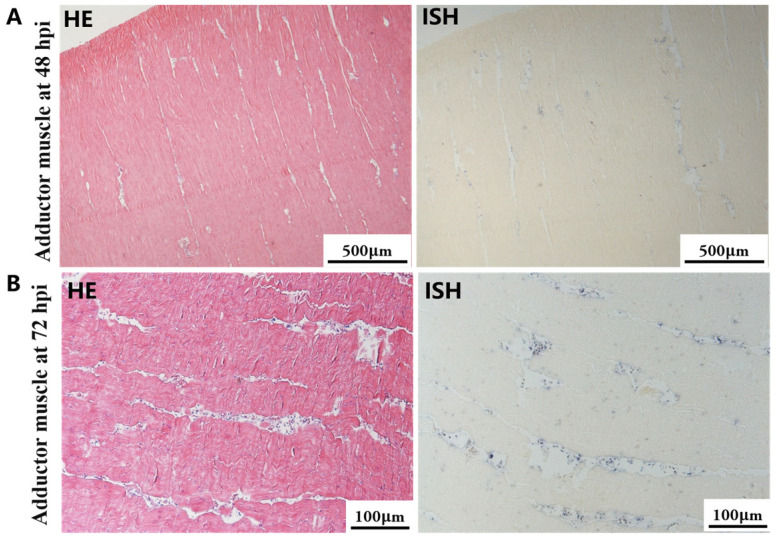
Histopathology and in situ hybridization investigation of OsHV-1 infected *A. broughtonii* adductor muscle. Positive results are indicated by black/blue precipitates. (**A**) Pathological changes (left) and positive cells (right) in the adductor muscle at 48 hpi. (**B**) Pathological changes (left) and positive cells (right) in the adductor muscle at 72 hpi. Scale bar = 500 μm and 100 μm, respectively.

The most notable sign of inflammation was the aggregation and infiltration of hemocytes in the connective tissues of the hepatopancreas and mantle, as well as in the gill filaments. The heavy infiltration of hemocytes was particularly evident around the vacuolated digestive tubules, and hybridization signals appeared at 48 hpi (Figure 3A). The most severe lesions associated with severe hemocyte infiltration and the strongest hybridization signals in the hepatopancreas were observed at 72 hpi (Figure 3B). At 48 hpi, the gill hemolymph vessels exhibited a pronounced repletion with OsHV-1 infected hemocytes (ISH signals, Figure 4A). While the repletion of hemocytes appears to have diminished at 72 hpi, there was an increase in the number of flat cells in the gill filaments that were infected by OsHV-1 (ISH signals, Figure 4B). The lesions observed in the tissue and the presence of viral DNA signals were predominantly found in the connective tissue and infiltrated cells. On occasion, OsHV-1 signals were also identified in fibroblast-like cells within the mantle (Figure 5A). Additionally, ISH-labeled cells were observed within the connective tissue of the mantle, especially along the muscle fibers (Figure 5B). In the foot and adductor muscle, muscle rupture and myonecrosis were observed with minimal or no hemocyte infiltration (Figure 6 and Figure 7). In addition to the detection of OsHV-1 DNA signals in infiltrated cells, the presence of these signals was also observed in the nuclei of cells dispersed within the muscle fibers.

## 4. Discussion

Mollusk farming represents one of the earliest and most significant forms of aquaculture, with mollusks being farmed on a global scale [34,35]. Despite the overall yield of mollusk aquaculture increasing at a rapid pace, the production of specific species and particular regions has suffered significant losses because of epidemic disease outbreaks [36,37,38,39,40,41]. Since its initial description in 1997, OsHV-1 has emerged as a significant concern within the mollusk aquaculture sector in China [42]. It has been confirmed that infections and associated mortalities have occurred in four bivalve species. The species of mollusks affected include the Chinese scallops *Chlamys farreri*, the Pacific oysters *C. gigas*, the blood clams *A. broughtonii*, and the half-crenated arks *Anadara kagoshimensis*, all of which are native to China [39]. Moreover, OsHV-1-specific positive PCR results have been documented in approximately a dozen additional species, although an active infection has not been conclusively established [43]. At present, the detection of OsHV-1 infection is predominantly reliant on molecular techniques, which encompass a range of PCR-based methods [44]. For example, quantitative PCR based on TaqMan^®^ and SYBR^®^ green chemistry, conventional PCR, propidium monoazide (PMA) real-time PCR, and LAMP have been employed in this context [29,45,46]. While molecular methods facilitate rapid detection and diagnosis, they are unable to ascertain an active infection or characterize the distribution of the virus-positive signal across multiple organs within the animal [47].

The most recent scientific advance associated with OsHV-1 infection has been primarily based on the molecular and biochemical approaches [48]. However, the mere detection of OsHV-1 and other pathogen DNA sequences in a cell–host does not necessarily indicate the establishment of an infection. It is advisable to corroborate the results of molecular analysis with those obtained through other techniques, such as histology and ISH, to ascertain their reliability [49]. The histological alterations associated with OsHV-1 infections lack specificity, rendering ISH a valuable approach for attaining a more profound comprehension of this disease. ISH is a technique that combines molecular cytology, histochemistry, and histology. It is a widely utilized complementary approach for virus detection and localization [49,50]. Infection with OsHV-1 has been diagnosed in several species using ISH, including *C. gigas* [30,51], *Ostrea edulis*, *Crassostrea angulata* [52], crustaceans (*Carcinus maenas*) and cephalopods (*Octopus vulgaris*) [9,10], while the physical and temporal distribution of OsHV-1 in *C. gigas* has been the subject of extensive study [30,51,53].

The cellular and tissue tropism, as well as the pathological characteristics of OsHV-1 in *A. broughtonii*, remain poorly understood. In this study, a foot injection of a viral suspension was employed in lieu of a cohabitation assay to simultaneously infect all blood clams with the same amount of OsHV-1 virus [28]. As reported by Xin et al. (2019) [28], OsHV-1 infection developed rapidly in *A. broughtonii*, with mortality rates reaching 95% at 72 h post-infection (hpi) and 100% at 96 hpi. High levels of OsHV-1 DNA load were observed in the mantle at 24 hpi, which was also the first appearance of mortality. A marked increase in both viral DNA (in the gill, foot, and adductor muscle) and clam mortality (from 5% to 55%) was observed between 24 and 48 h post-infection. The viral DNA load reached its maximum concentration in the foot and adductor muscle at 48 hpi, and in the gill, mantle, and hepatopancreas at 72 hpi. The C2/C6 probe was utilized to identify positive virus signals in the challenge group through in situ hybridization (ISH), thereby enabling the tracing and localization of OsHV-1 in five distinct tissues of *A. broughtonii*. At 6~24 hpi, low viral DNA detection by qPCR was positive, whereas viral DNA detection on histological sections by ISH was negative for blood clams *A. broughtonii* infected with OsHV-1. These results may be partially attributed to the enhanced sensitivity of qPCR in comparison to ISH, given that the former involves a replication step of genetic material, whereas the latter does not [54]. ISH signals of OsHV-1 were observed in the mantle, hepatopancreas, gill, foot, and adductor muscle at 48 hpi, concomitant with an increase in the viral DNA load above 10^2^ copies/ng total DNA. Nevertheless, it should be noted that a one-to-one correspondence between viral DNA loads detected by qPCR and ISH results is not absolute. For instance, no ISH signals were discerned in the mantle collected at 24 hpi with a high viral DNA load (4.46 × 10^4^ copies/ng total DNA), whereas ISH signals were identified in the adductor muscle collected at 72 hpi with a low viral DNA load (3.46 copies/ng total DNA). It is postulated that sampling bias during DNA extraction and qPCR (less than 30 mg of tissue were sampled) may be partially responsible for the inconsistent results between ISH and qPCR.

It is reasonable to find that OsHV-1 DNA loads increased with the development of infection after experimental infection [55,56], while the decrease or sudden drop of the viral loads at the end of the viral disease has also been frequently reported both in *C. gigas* and *A. broughtonii* [28,57,58]. Similar infection process and mortality patterns with the present study were reported in Xin et al. (2019); they revealed that a decrease in the amount of hemocytes were evidenced in the infected *A. broughtonii* from 48 hpi to 72 hpi [28]. In the present study, a sudden drop and decrease in OsHV-1 DNA loads were revealed in adductor muscle and foot at 72 hpi, but not in the gill, mantle and hepatopancreas. We speculate that the depletion of hemocytes as the infection advances, and the lack of connective tissues in the adductor muscle and foot, should be responsible for the decrease in the viral DNA loads. As reported previously in *C. gigas* and *A. broughtonii* [28,51,59,60], fluctuations of the viral loads at the initial stage (12 hpi in the mantle) of viral disease have also been found in the present study. These results suggested that the immune system of clams was trying to manage the viral infection and control its replication.

Studies on OsHV-1 infection in *C. gigas* have indicated that the connective tissue of various organs, including the mantle, gill and digestive gland, is the primary target of OsHV-1 infection. This finding is supported by the literature, as evidenced by references [32,51,52]. Additionally, fibroblastic-like cells, hemocytes, and myocytes have been demonstrated to be susceptible to OsHV-1 infection [31,51]. In the present study, a substantial number of hemocytes and associated ISH signals were also observed in the hepatopancreas and gills (see Figure 3 and Figure 4). Additionally, a substantial number of fibroblast-like cells within the connective tissues of the mantle were identified and labeled with ISH signals (Figure 5). The histological lesions were invariably accompanied by abnormal nuclei, which exhibited either marginated chromatin or pyknosis. However, cellular infiltration was only observed in a subset of cases [30,51]. Additionally, OsHV-1-specific probe labeling revealed the presence of infected nervous cells throughout the visceral ganglion in infected oyster spat sections. Furthermore, labeled cells were observed in the gonads of adult oysters, some of which were identified as oocytes [61,62]. These findings indicate the potential for vertical transmission of OsHV-1 through the infected gametes of asymptomatic adults [30,52]. Furthermore, intranuclear acidophilic inclusions comparable to Cowdry type A inclusions were identified in some oyster species infected with OsHV-1, including *Ostrea edulis* [63,64] and *Ostrea angasi* [65], but not in *C. gigas*. The present study did not detect Cowdry type A inclusions. On occasion, weak ISH signals were observed in the nuclei of potential myocytes in the foot and adductor muscles (see Figure 6 and Figure 7).

## 5. Conclusions

Since the initial characterization of OsHV-1 infection in *A. broughtonii* in 2012, this pathogen has emerged as the primary etiology responsible for mass mortalities in the species. A substantial body of scientific literature has emerged on the characterization of the viral disease. The objective of this study was to characterize the pathological changes and tissue tropism during the development of OsHV-1 infection in *A. broughtonii*. The results demonstrated that hemocytes and fibroblastic-like cells were the primary cellular targets of OsHV-1. Additionally, lesions, infiltrated hemocytes, and co-localized ISH signals were identified in muscular tissues of the foot and adductor muscle. These findings contribute to the understanding of OsHV-1 pathogenesis in Arcidae mollusks.

## Figures and Tables

**Table 1:** The OsHV-1 DNA detected in experimentally infected *Anadara broughtonii* by qPCR and in situ hybridization.

Table (hpi)	Mantle		Hepatopancreas		Gill		Foot		Adductor Muscle	
	ISH	qPCR	ISH	qPCR	ISH	qPCR	ISH	qPCR	ISH	qPCR
0	-	1.13	-	5.26	-	5.70	-	4.94	-	5.54
6	-	1.89 × 10^1^	-	5.38	-	5.35	-	5.06	-	5.00
12	-	1.57 × 10^1^	-	5.14	-	5.63	-	5.69	-	5.42
24	-	4.46 × 10^4^	-	6.27	-	6.60	-	5.02	-	6.22
48	+	4.98 × 10^4^	++	1.14 × 10^1^	+	2.67 × 10^2^	++	2.87 × 10^2^	++	3.91 × 10^3^
72	++	5.45 × 10^5^	++	5.08 × 10^5^	++	5.03 × 10^5^	++	2.54 × 10^2^	+	3.46

The “++”, “+” and “-” symbols indicate the presence of a high-intensity positive signal, a low-intensity positive signal, and the absence of a positive signal, respectively. The abbreviation “hpi” stands for “hours post injection”.

## Data Availability

The original contributions presented in the study are included in the article; further inquiries can be directed to the corresponding author.

## Supplementary Materials

The following supporting information can be downloaded at: https://www.mdpi.com/article/10.3390/biology13090720/s1, Figure S1: The original gel image of Figure 2 in the main text.
