# Supplementary material for: Localization and Tissue Tropism of Ostreid Herpesvirus 1 in Blood Clam Anadara broughtonii
Source: Biology (Basel). 2024 Sep 13;13(9):720. doi: 10.3390/biology13090720 (PMC11429395; doi:10.3390/biology13090720)
Supplement: Supplementary file 1 [file biology-13-00720-s001.zip › biology-3188757-supplementary.pdf]

SUPPLEMENTARY INFORMATION

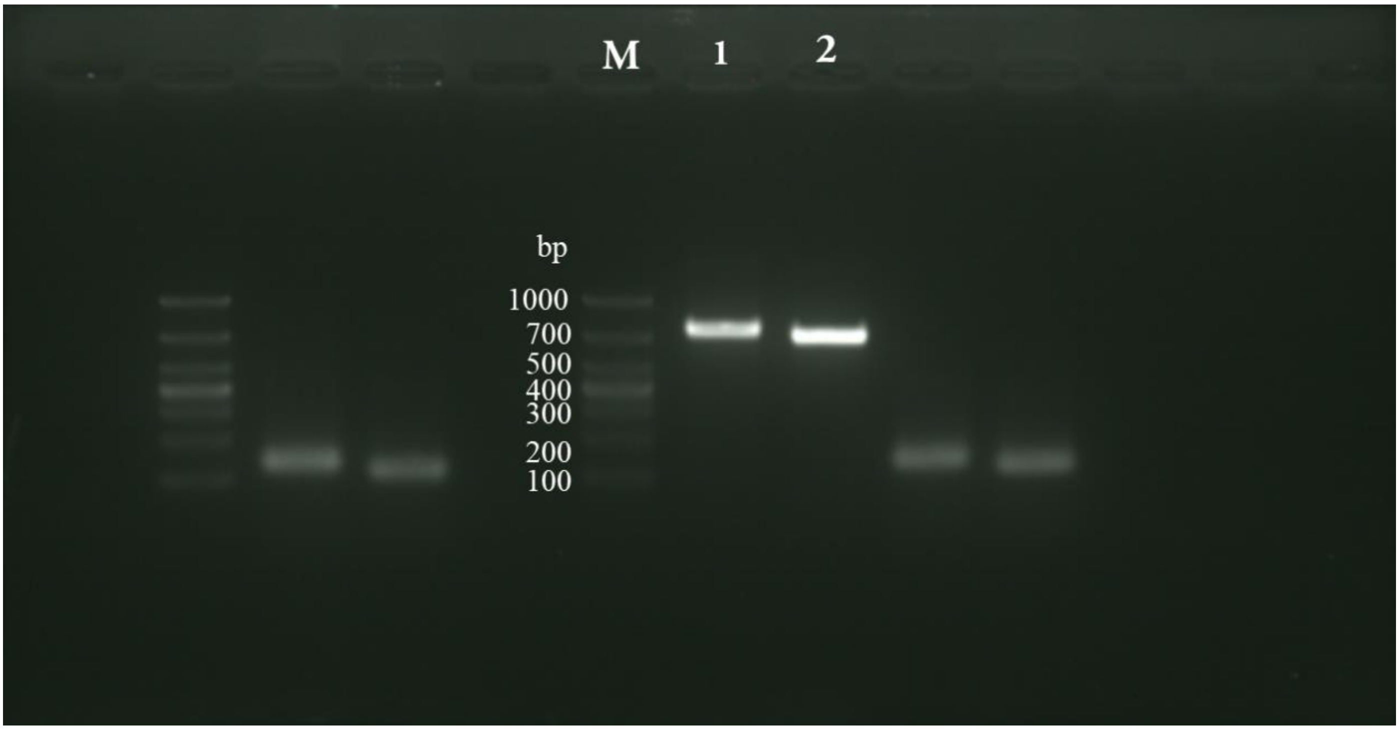

Figure S1. The original gel image of Figure 2 in the main text.

M: DNA marker. Line 1 indicated digoxigenin (DIG) -dUTP were incorporated into the amplification products during specific PCR amplification of OsHV-1, therefore the molecular weight of the PCR product is higher than the amplification products of common PCR in Line 2. The bands in the other lines were not related to the present study.
